# Extracellular cyclophilin-A stimulates ERK1/2 phosphorylation in a cell-dependent manner but broadly stimulates nuclear factor kappa B

**DOI:** 10.1186/1475-2867-12-19

**Published:** 2012-07-04

**Authors:** Karim Bahmed, Curtis Henry, Michael Holliday, Jasmina Redzic, Madalina Ciobanu, Fengli Zhang, Colin Weekes, Robert Sclafani, James DeGregori, Elan Eisenmesser

**Affiliations:** 1 Department of Biochemistry and Molecular Genetics, School of Medicine, University of Colorado Denver, Aurora, CO, 80045, USA; 2 Department of Medicine, Division of Oncology, School of Medicine, University of Colorado Denver, Aurora, CO, 80045, USA; 3 National High Magnetic Field Laboratory, Tallahassee, FL, 32310, USA; 4 12801 E 17th Ave, Aurora, CO, 80045, USA

**Keywords:** Extracellular cyclophilin-A, PPIA, BSG, EMMPRIN, CD147, MMP, Cytokine, Interleukins, Pancreatic cancer, Leukemia

## Abstract

**Background:**

Although the peptidyl-prolyl isomerase, cyclophilin-A (peptidyl-prolyl isomerase, PPIA), has been studied for decades in the context of its intracellular functions, its extracellular roles as a major contributor to both inflammation and multiple cancers have more recently emerged. A wide range of activities have been ascribed to extracellular PPIA that include induction of cytokine and matrix metalloproteinase (MMP) secretion, which potentially underlie its roles in inflammation and tumorigenesis. However, there have been conflicting reports as to which particular signaling events are under extracellular PPIA regulation, which may be due to either cell-dependent responses and/or the use of commercial preparations recently shown to be highly impure.

**Methods:**

We have produced and validated the purity of recombinant PPIA in order to subject it to a comparative analysis between different cell types. Specifically, we have used a combination of multiple methods such as luciferase reporter screens, translocation assays, phosphorylation assays, and nuclear magnetic resonance to compare extracellular PPIA activities in several different cell lines that included epithelial and monocytic cells.

**Results:**

Our findings have revealed that extracellular PPIA activity is cell type-dependent and that PPIA signals via multiple cellular receptors beyond the single transmembrane receptor previously identified, Extracellular Matrix MetalloPRoteinase Inducer (EMMPRIN). Finally, while our studies provide important insight into the cell-specific responses, they also indicate that there are consistent responses such as nuclear factor kappa B (NFκB) signaling induced in all cell lines tested.

**Conclusions:**

We conclude that although extracellular PPIA activates several common pathways, it also targets different receptors in different cell types, resulting in a complex, integrated signaling network that is cell type-specific.

## Background

Cyclophilins (Cyps) are members of the immunophilin family of enzymes that possess peptidyl-prolyl isomerase (PPIase) activity whereby they catalyze the reversible cis/trans interconversion of the imide bond in proline residues. Cyps are implicated in promoting different diseases and involved in diverse pathological conditions including human immunodeficiency virus infection [1], hepatitis C infection [2], arteriosclerosis [3], inflammation [4], and multiple cancers [5]. The prototypical cyclophilin family member, cyclophilin-A (PPIA), was initially discovered as the intracellular target of cyclosporin-A over a quarter century ago, yet only recently have the extracellular roles of PPIA in inflammatory disorders and in driving dozens of cancers emerged [6]. In fact, PPIA is now commonly referred to as a cytokine [7,8]. Unlike several Cyps, PPIA does not contain a signal peptide and is therefore secreted by noncanonical mechanisms that include vesicular release [9]. Many clinical reports have identified high levels of extracellular PPIA in synovial fluids of rheumatoid arthritis patients [10] and elevated levels in the peripheral blood of cancer patients relative to healthy individuals [4]. Moreover, increased expression of PPIA leads to chemotherapy resistance in multiple cancers [11] and RNA interference (RNAi) of PPIA in a non-small cell lung cancer model slowed tumor growth [12], thereby suggesting a causative role in cancer progression. Such findings illustrate the potential of therapeutically targeting extracellular PPIA but also the downstream events that PPIA may regulate, which is dependent on further characterization of extracellular PPIA activities studied here.

The particular cellular targets of extracellular PPIA and downstream signaling pathways have recently been explored; however, these studies have often been met with contradictory findings. For example, extracellular PPIA has been shown to induce JNK and p38 phospho-rylation in some studies but not in others [7,13,14]. Some of these detected differences may reflect differential responses from different cell types, i.e., endothelial cells versus monocytes for JNK and p38. However, much of this work has been further complicated by the findings of Payeli et al. [15], which exposed the wide-spread use of impure commercially purchased recombinant PPIA preparations. Further confusion has centered on the role of PPIA catalytic activity (i.e., cis/trans isomerization) on its biological activity. For example, elegant biophysical studies have shown that the isomerase activity of intracellular PPIA regulates the cis/trans conformation state of several biological targets, which in turn regulate their downstream interactions [16]. Analogous to this, we have shown that extracellular PPIA can catalyze isomerization of the only known cellular protein receptor, Extracellular Matrix MetalloProteinase Inducer (EMMPRIN), also known as CD147 and Basigin, and that this occurs at the membrane proximal region centered around EMMPRIN Pro211 [17]. However, we also previously discovered that the PPIA/EMMPRIN interaction is extremely weak in vitro, on the order of millimolar affinity, potentially alluding to the requirement of other unknown mediators or other cellular targets of PPIA.

In this study we utilized highly purified recombinant PPIA to probe its extracellular activities on multiple cell lines that included both leukemic and pancreatic cancer cells [18]. These particular cell lines were chosen since they represent very different types of cancers, i.e., monocytic leukemia cells and epithelial cells in pancreatic cancer, therefore allowing us to probe the particular similarities and differences that extracellular PPIA may regulate in a range of cancers. Initial screens that employed over a dozen luciferase reporters were followed with dose-dependent luciferase reporter assays and other intracellular signaling assays to probe ERK1/2 phosphorylation and NFκB signaling that are all mediated by extracellular PPIA. Moreover, by utilizing mutations on both the PPIA protein side as well as within the promoter regions of the luciferase reporters, specificity was shown and downstream promoter elements were identified. Collectively, our systematic study indicates that while some of the final activated transcription events mediated by extracellular PPIA stimulation are shared (i.e., NFκB signaling), there are cell-specific differences in activation networks (i.e., ERK1/2 phosphorylation), which may be reliant on multiple receptors beyond the cellular EMMPRIN receptor.

## Results

### Recombinant purified PPIA is identical to that previously described

Based on the work conducted by Payeli et al. [15], it is critical for any study that monitors the biological effects of recombinant PPIA to first show that this enzyme is of the highest purity. Specifically, these authors took advantage of the uniquely characteristic UV spectrum of PPIA and have shown that much of the commercially available PPIA used is highly impure. Thus, to investigate the biological functions of extracellular PPIA, we purified recombinant PPIA as we have recently described utilizing a rigorous procedure that encompasses two ion-exchange columns and a sizing column [17,18]. The endotoxin levels of these recombinant preparations are identical to that of the purified water used (<30 pg/ml) and the UV spectrum is identical to the pure PPIA (Additional file 1: Figure S1) that has been previously described by Payeli et al.

### Extracellular PPIA stimulation leads to a wide range of cellular responses

**Figure 1:**
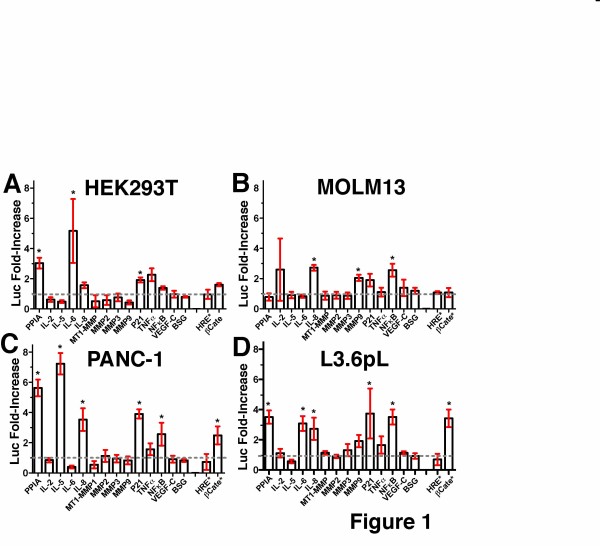
**Extracellular PPIA activity monitored through luciferase reporters assays.** Sixteen luciferase reporters were used to monitor the response to extracellular PPIA in several cell lines, which included the following: **A**) HEK293T cells, **B**) MOLM13 cells, **C**) PANC-1 cells, and **D**) L3.6PL. Cells were transiently transfected with the indicated reporters and then stimulated with recombinant 25 μM PPIA as described in Materials and Methods. Luciferase activity is shown as a fold-increase over the buffer control (Luc Fold-Increase). Dashed line (grey) indicate no Luc Fold-Increase (i.e., unity). *Beta-Catenin (βCate) and *HRE refer to plasmids that comprise the transcription target sites for HIF and βCate transcription factors, respectively. The remaining plasmids comprise the indicated promoters for each protein. Bars indicate the standard error of two replicates.

A comparative analysis of multiple cell lines was conducted using luciferase reporter assays, since such assays offer a highly sensitive quantitative analysis of the signaling events under extracellular PPIA regulation. Initial screens of 16 luciferase reporters were monitored with recombinant PPIA and used with several cell lines that included standard a model cell line - HEK293T cells (Figure 1A), a monocytic leukemia cell line - MOLM13 (Figure 1B), a pancreatic cancer cell line - PANC-1 (Figure 1C), and a metastatic pancreatic cancer cell line - L3.6pL (Figure 1D). The luciferase reporters used encode for the promoters of multiple cytokines and matrix metalloproteinases (MMPs), but also several binding elements that directly report on the activity of the transcription factors themselves such as hypoxia-responsive element (HRE) and β-catenin binding element (βCate). These report on hypoxia-inducible factor (HIF) and β-catenin activities, respectively. The particular luciferase reporters were chosen since their respective cytokines, MMPs, and transcription factors have been implicated in both inflammation, tumorigenesis and metastasis and the chosen PPIA concentration was based on the relatively high levels of extracellular PPIA found in diseased tissue [6,10].

Both cell-specific and common responses were found to be mediated by extracellular PPIA (Figure 1). For example, since PPIA has previously been shown to induce an increase in Interleukin (IL)-5 production in PANC-1 cells [19], we sought to determine whether a similar response could be induced in other cell types. However, despite confirming that PPIA induced a relatively high increase in IL-5 in PANC-1 cells (Figure 1C), there was no similar response detected in any of the other cell lines that included the other pancreatic cancer cell line, L3.6pL (Figure 1A,B,D). The addition of extracellular PPIA induced a luciferase reporter activity for PPIA itself in all cell lines tested excluding MOLM13, consistent with the role of increased intracellular PPIA in malignant transformation [20]. PPIA induced MMP9 luciferase reporter activity in both MOLM13 and L3.6pL cell lines. MMP9 production appears to be a general response to extracellular PPIA in monocytic cell lines such as MOLM13 as it has been observed in THP-1 cells as well [21,22]. Previous studies utilizing commercially purchased recombinant PPIA have suggested that PPIA stimulates IL-6 in monocytic cells [23]. However, our recombinant PPIA did not induce the production of IL-6 in the monocytic MOLM13 cells, which is analogous to the findings of Payeli et al. that have also shown that monocytic cells do not produce IL-6 in response to highly purified PPIA [15]. There are several reporters that appear to be universally upregulated by extracellular PPIA, which include NFκB, IL-8, and p21^Cip1^ (referred to herein as p21). The general response of NFκB and IL-8 was further confirmed by multiple experiments below, while the uniform increase in p21 levels observed for all cell lines could suggest a noncanonical role of this cyclin kinase inhibitor (CKI) in cancer progression. Consistent with such a noncanonical role, PPIA did not induce cell-cycle arrest in any of these cell lines within the same 24 h time period used for the luciferase based assays (Additional file 2: Figure S2). In fact, an increase in p21 protein has previously been found to correlate to pancreatic cancer progression [24] and the complicated array of post-transcriptional modifications to p21 have been found to promote cell survival and not always cell-cycle arrest [25].

Concerns regarding the commercial preparations of recombinant PPIA have recently emerged [15] and therefore, here we addressed both the purity and specificity of recombinant PPIA by using several other cytokines. Specifically, IL-6 and IL-8 were recombinantly produced and purified since, like PPIA, these cytokines are involved in both inflammation and cancer progression [26,27]. The specific response induced by recombinant PPIA was shown by a comparative analysis with recombinant IL-6 where a differential response was observed in regard to both MMP9 and p21 stimulation (Additional file 3: Figure S3A). Like PPIA, IL-6 stimulated the activity of luciferase reporters of IL-8 and NFκB, which is consistent with previous observations of monocytes [28]. We also showed that there is a cell-specific response between IL-6 and IL-8 in regard to inducing PPIA luciferase activity (Additional file 3: Figure S3B), showing that these two interleukins that were purified similarly exhibit differential activity rather than inducing a general effect. Further confirmation of specificity was also indicated by a significantly diminished response to a PPIA active site mutant in all cell lines tested, the details of which are presented below. Thus, these comparative studies confirm that the recombinant proteins utilized here induce specific cellular responses.

### PPIA activity is dose-dependent and partially reliant on the PPIA active site

**Figure 2:**
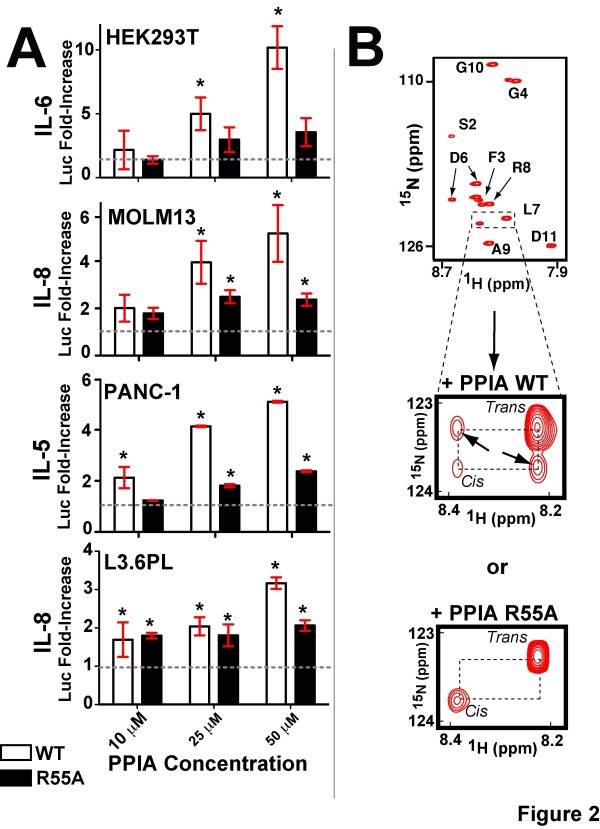
**Probing the involvement of the PPIA active site for both its biological activity and catalytic activity. A**) For PPIA biologically activity, dose-dependent luciferase assays were conducted as in Figure 1 for the most highly induced luciferase reporter in each cell line using both wild type PPIA (white) and the active site point mutation PPIA R55A (black). These include IL-6 in HEK293T cells, IL-8 in MOLM13 cells, IL-5 in PANC-1 cells, and IL-8 in L3.6pL cells. **B**) The ^15^N-HSQC spectrum of 1 mM ^15^N-labeled model peptide substrate, GSFGPLRAGD, is shown alone with the associated assignments for each amide (top). Note, two resonances are observed corresponding to the slowly interconverting cis and trans resonances. Catalysis of both the wild type PPIA (middle) and mutant PPIA R55A (bottom) were assessed through ZZ-exchange spectroscopy in the presence of 20 μM of each. ZZ-exchange spectra are shown for the same 240 ms delay. Arrows denote the appearance of exchange peaks due to PPIA-mediated enhancement of the rate of cis/trans interconversion of the model peptide substrate. We note that no exchange peaks were observed for longer mixing times of PPIA R55A indicating no detectable catalytic rate enhancement. All spectra were collected at 720 MHz at 10 C.

The most highly upregulated luciferase reporter induced by PPIA within each cell line in Figure 1 was further probed in a dose-dependent manner (Figure 2A, white). A dose–response using recombinant PPIA was observed in each cell line, which included IL-6 in HEK293T cells, IL-8 in MOLM13 cells, IL-5 in PANC-1 cells, and IL-8 in L3.6pL cells (Figure 2A). Such a dose–response over this range of micromolar PPIA concentrations indicates that the overall binding constant of extracellular PPIA is weak in nature. This is consistent with previous reports that have described micromolar affinities of PPIA to biological targets such as the HIV-1 capsid protein [29] and peptides derived from phage display libraries [30]. The weak affinity of PPIA interactions has made the identification of PPIA binding partners notoriously difficult via standard pull-down methods [31]. However, the interaction is specific as indicated by the diminished response to the active site mutant, PPIA R55A (Figure 2A, black). This PPIA R55A point mutation has been reported to comprise only 1 % of the enzymatic acti-vity (i.e., PPIase activity) compared to the wild type enzyme [32]. Therefore, an observed reduction in biological activity for PPIA R55A relative to the wild type enzyme is often used to indicate a role of enzymatic activity in biological activity. However, caution must be taken in such a conclusion since this same mutant engages substrates with an approximately 10-fold lower affinity than the wild type enzyme as well [33], thereby making it difficult to decouple the role of PPIA binding versus PPIA catalysis in biological activity. Nonetheless, a comparative analysis of PPIA R55A to that of the wild type PPIA indicates that the biological activity in all cell lines is markedly reduced (Figure 2A), confirming an important role of the PPIA active site.

One potential concern is that the endogenous bacterial cyclophilins may co-purify with the recombinant human PPIA that is overexpressed, which was addressed here. We wanted to address this since there remained some residual activity in all cell lines tested for the PPIA R55A mutant (Figure 2A), suggesting that a non catalytic role for extracellular PPIA may also be present. To probe enzymatic activity and confirm no catalytic activity for PPIA R55A, an ^15^N-labeled peptide was recombinantly produced as we have previously described in order to apply a highly sensitive NMR-based catalytic assay called ZZ-exchange [17]. ZZ-exchange is ideal for quantifying the rate of proline isomerization mediated by cyclophilins as we have previously described [17,34] and the peptide sequence, GSFGPDLRAGD, was based on a previous phage display study for CypA [30]. For the ^15^N-labeled peptide alone, both the cis and trans conformations are observed for several resonances (Figure 2B, top), indicating two distinct chemical environments with a slow uncatalyzed cis/trans interconversion of the central proline as observed for other PPIA substrates [17,35]. However, in the presence of catalytic concentrations of PPIA the appearance of “exchange peaks” within ZZ-exchange spectra of ^15^N-labeled substrate indicate that PPIA enhances the inherently slow peptidyl-prolyl isomerization (Figure 2B, middle). As expected, in the presence of the active site mutant, PPIA R55A, there are no detectable exchange peaks for the substrate and therefore indicates that the catalytic efficiency is severely impaired. Importantly, this atomic resolution description also confirms the absence of a co-purified bacterial cyclophilin that may be responsible for the residual biological activity observed for PPIA R55A. Therefore, there may exist some interactions for extracellular PPIA that either do not require the enzymatic activity of PPIA or simply do not require the PPIA active site.

### Extracellular PPIA activates IL-6 and IL-8 interleukins via NFκB for multiple cell lines

Since many promoters that include proinflammatory cytokines, such as IL-6 and IL-8, comprise NFκB binding elements, we hypothesized that the NFκB pathway is involved in this translational activation. This hypothesis was initially prompted by our luciferase reporter screens (Figure 1), yet was further tested here through several experimental approaches.

**Figure 3:**
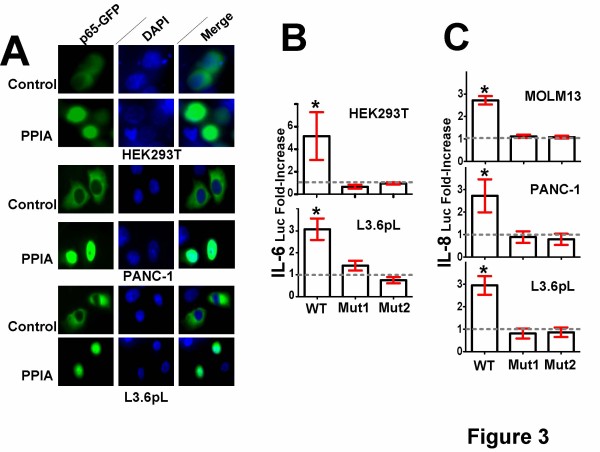
**Characterizing extracellular PPIA-mediated activity of NFκB within multiple cell lines. A**) An NFκB translocation assay was conducted in several adherent cell lines, which included HEK293T cells (top), PANC-1 cells (middle), and L3.6pL cells (bottom) with the addition of either buffer control or PPIA. Cells were transiently transfected with the p65-GFP plasmid (green, left panels) as well as DAPI stained (blue, middle panels) and the merge is shown (green, right panels). **B**) PPIA-induced luciferase reporter activity was monitored for the wild type IL-6 reporter (WT) and two mutations to the NFκB binding element (Mut1 and Mut2) for HEK293T and L3.6pL cells. **C**) PPIA-induced luciferase reporter activity was monitored for the wild type IL-8 luciferase reporter (WT) and two mutations within the NFκB binding element (also called Mut1 and Mut2) for MOLM13, PANC-1, and L3.6pL cells. PPIA-induced luciferase reporter activity was monitored as in Figure 1.

For the first method, we examined the effect of extracellular PPIA on NFκB translocation from the cytoplasm to the nucleus. To examine translocation we employed an elegant assay that monitors the fluorescence of a p65 chimera C-terminally tagged to the green fluorescent protein (GFP) previously established by Schmid et al. [36,37]. Specifically, all three adherent cell lines studied here were transfected with a plasmid encoding for GFP-p65, which included HEK293T cells (Figure 3A, top), PANC-1 cells (Figure 3A, middle), and L3.6pL cells (Figure 3A, bottom). Analogous to the findings of Schmid et al. [37], which showed that TNFα induced GFP-p65 translocation to the nucleus, treatment here with PPIA also induced translocation in all three cell lines (Figure 3A, bottom panels) whereas the control of buffer alone did not (Figure 3A, top panels). Moreover, even the mutant PPIA R55A was capable of inducing GFP-p65 translocation (Additional file 4: Figure S4A), consistent with the residual activity observed for this active site mutant with regard to luciferase activity of reporter genes (Figure 2A). While these results imply that extracellular PPIA induces IkB degra-dation in order to allow for GFP-p65 translocation, we confirmed this by direct visualization using immunocytofluorescence (see Additional file 4: Figure S4B). Specifically, addition of PPIA led to a decreased immunocytofluorescence signal for the specific IkBα isoform (Additional file 4: Figure S4B, bottom panels) while buffer alone did not (Additional file 4: Figure S4B, top panels). Thus, from these data, we clearly show that extracellular PPIA is capable of NFκB activation in all three adherent cell lines tested.

As a second approach, we confirmed that the NFκB binding element is necessary for PPIA induction of luciferase reporters for both IL-6 and IL-8. Specifically, we utilized mutations to the NFκB binding element within the IL-6 promoter that had previously been shown to abrogate IL-6 luciferase reporter activity induced by the potent inflammatory peptide, bradykinin [38]. These NFκB promoter mutations are referred to as Mut1 and Mut2 as described in Materials and Methods. Since both HEK293T and L3.6pL cells were positive for the IL-6 luciferase reporter (Figure 1A, D), these IL-6 promoter mutants were tested in parallel to the wild type IL-6 luciferase reporter. Analogous to bradykinin, PPIA stimulation of IL-6 was also found to be reliant on the NFκB binding element as shown by the abrogation of luciferase reporter activity in both HEK293T and L3.6pL cells (Figure 3B). Additionally, two similar mutations were made to the NFκB binding element within the IL-8 promoter as described in Materials and Methods and the analogous experiments were conducted in MOLM13, PANC-1, and L3.6pL cells. These cell lines were chosen since they all exhibited approximately a 3-fold increase in IL-8 luciferase reporter activity upon stimulation with recombinant PPIA in Figure 1. Just as mutations to the NFκB binding element within the IL-6 promoter abrogate a PPIA driven increase in the IL-6 luciferase reporter activity, mutations to the NFκB binding element within the IL-8 promoter lead to a negligible response to PPIA (Figure 3C). Thus, the NFκB binding element is required for extracellular PPIA stimulation of multiple cytokines (i.e., IL-6 and IL-8).

### ERK1/2 phosphorylation mediated by extracellular PPIA is cell dependent

**Figure 4:**
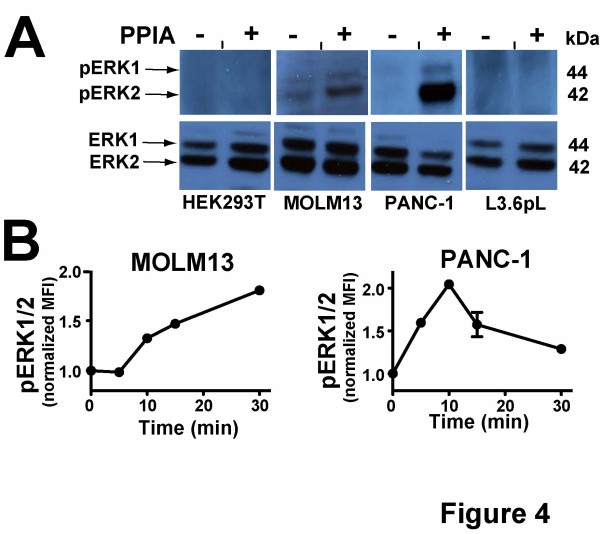
**Characterizing extracellular PPIA-mediated activation of ERK1/2 phosphorylation within multiple cell lines. A**) ERK1/2 phosphorylation in PPIA stimulated HEK293T, MOLM13, PANC-1, and L3.6pL cells was determined by immunoblotting. Cells were treated with either buffer alone (−) or 25 μM PPIA (+) for 10 min and stored in SDS loading buffer for subsequent immunoblotting analysis of pERK1/2 and total ERK1/2 as a control. **B**) PPIA-induced time-dependent induction of ERK1/2 phosphorylation as monitored by flow cytometry is shown for both MOLM13 and PANC-1 cells for the indicated time points also stimulated with 25 μM PPIA. The data shown represents the mean of fluorescence intensity (MFI) for each time point measured in triplicate and normalized to the average initial MFI.

To further investigate pathways downstream of extracellular PPIA stimulation, we compared ERK1/2 phosphory-lation in multiple cell lines (Figure 4). There has been some precedence for extracellular PPIA-mediated ERK1/2 phosphorylation in several cell lines that include monocytes, macrophages, neuronal, and lung cancer epithelial cells [13,14,22,23,39] and deregulation of ERK1/2 signaling is implicated in many diseases including cancer [40]. Thus, the same four cell lines used for screening luciferase reporters in Figure 1 were also used to monitor PPIA-mediated ERK1/2 phosphorylation (Figure 4A).

ERK1/2 activation was initially assessed with the addition of 25 μM PPIA using western blot analysis and then more quantitatively probed using a cytometric assay (Figure 4). While no phosphorylation was observed in either HEK293T or L3.6pL cells, phosphorylation was observed for both MOLM13 and PANC-1 cells (Figure 4A). Further analysis using a flow cytometric assay described in Materials and Methods indicated a characteristic timeframe for the increase in ERK1/2 phosphorylation for both MOLM13 and PANC-1 cells (Figure 4B). No time-dependent change was observed for HEK293T or L3.6pL cells in accord with the western blot analysis (data not shown). It is important to note that although the classical pathways that lead to ERK1/2 phosphorylation and NFκB transcription probed above are independent, there are some studies that have shown that these two pathways exhibit some cross-talk [13,22,23,41]. However, the data presented here show that PPIA stimulated NFκB activity in all cell lines tested (Figure 3) while only a subset of these cell lines exhibit an increase in ERK1/2 phosphorylation (Figure 4), which also suggests that the two pathways may be independent. The important point here is that extracellular PPIA induces ERK1/2 phosphorylation in a cell-dependent manner and, once again, there is also a differential response within cell lines derived from the same type of cancer (i.e., PANC-1 and L3.6pL cells).

### Extracellular PPIA activity is reliant on cellular BSG as well as other receptors

Much attention has focused on the interaction between extracellular PPIA and its proposed cellular receptor, BSG, prompting us to determine whether a PPIA/BSG interaction may be critical for some of the observed activities above [6]. Our overall results confirm that cellular BSG is involved in some signal transduction events but also implicate other as yet unknown receptors that are targeted by extracellular PPIA.

**Figure 5:**
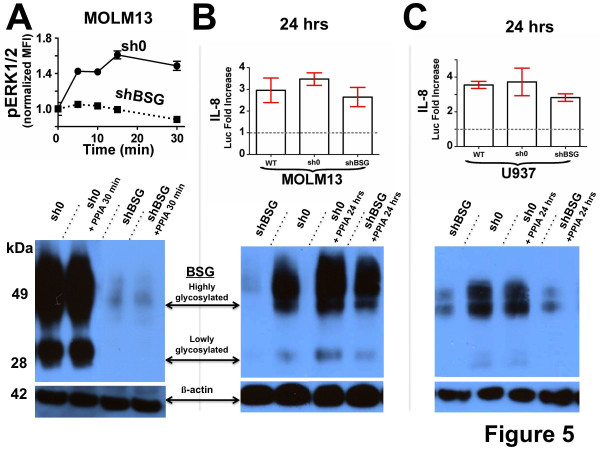
**Assessing the importance of cellular BSG for extracellular PPIA activity on monocytes. A**) Top: ERK1/2 phosphorylation was monitored in MOLM13 cells using a flow cytometric assay (as in Figure 4) for both sh0 cells (solid line) and shBSG cells (dashed line). Bottom: Western blot analysis using the BSG antibody shows that shBSG cells exhibit a significant knockdown when compared to sh0 cells. No significant increase in cellular BSG expression was observed in sh0 cells within this 30 min timeframe as assessed by western blot analysis. **B**) Top: In MOLM13 cells, IL-8 luciferase reporter activity was monitored after stimulation with recombinant PPIA. Bottom: Cellular BSG was monitored via western blot analysis both before and after 24 h stimulation with recombinant PPIA. **C**) In U937 cells, a similar analysis was conducted as in (**B**). For all stimulations, 25 μM recombinant PPIA was used and β-actin was used as a loading control. Arrows denote both highly and lowly glycosylated BSG as well as the β-actin loading control.

Our initial studies focused on determining whether cellular BSG is important for the extracellular PPIA-mediated phosphorylation of ERK1/2 in the MOLM13 monocytic cell line observed in Figure 4. To this end, we utilized a specific shRNA BSG knockdown (shBSG) as well as a control cell line with a non coding shRNA knockdown (sh0) to compare PPIA-induced ERK1/2 phosphorylation in a time-dependent manner (Figure 5A, top). From densitometry, the shBSG knockdown compared to sh0 in MOLM13 cells was determined to be approximately 94 % (Figure 5A, bottom). As expected, knockdown of BSG in MOLM13 cells resulted in a drastic decrease in PPIA-mediated ERK1/2 phosphorylation. To account for the possibility that PPIA may induce BSG cell surface expression within the timeframe of the ERK1/2 phosphorylation assay, we also monitored cellular BSG within the same 30 min timeframe and found no detectable increase in BSG expression in MOLM13 cells (Figure 5A, bottom), which is consistent with the lack of PPIA-induced ERK1/2 phosphorylation. Cellular BSG has been shown to be cri-tical for PPIA-mediated phosphorylation of ERK1/2 in several other cell lines such as HeLa cells [42] and lung cancer cells [14]. Thus, our data here clearly shows that cellular BSG is also critical for PPIA-mediated ERK1/2 phosphorylation in MOLM13 cells.

We next determined whether cellular BSG is important for an activity measured in our luciferase assays. We focused on PPIA-mediated upregulation of IL-8, since this cytokine is a well-known contributor to inflammation and cancer progression [27]. Knockdown of cellular BSG had a negligible effect on the activity of PPIA-mediated upregulation of IL-8 reporter activity in MOLM13 cells (Figure 5B, top). However, PPIA also induced cellular expression of BSG within MOLM13 cells that was clearly visible within the shBSG knockdown cells after the 24 h period used to monitor the luciferase reporter (Figure 5B, bottom). Thus, because there is simultaneous re-expression of cellular BSG upon stimulation with recombinant PPIA, BSG’s role in PPIA-mediated IL-8 upregulation cannot be determined in MOLM13 cells. Therefore, we turned to another monocytic cell line, U937 cells, and produced the analogous stable knockdown cell lines produced in MOLM13 cells. From densitometry, the shBSG knockdown compared to sh0 in U937 cells was determined to be approximately 83 % (Figure 5C, bottom). Interestingly, cellular BSG was also dispensable for PPIA-mediated induction of IL-8 in U937 cells as IL-8 induction was unchanged between shBSG and sh0 cells (Figure 5C, top) and no concomitant induction of cellular BSG within the same 24 h period was observed (Figure 5C, bottom). Thus, our studies here have shown that in at least some cell lines, extracellular PPIA stimulates IL-8 through another receptor other than BSG and that PPIA stimulation of cellular BSG expression is cell-dependent.

### PPIA binds heparin through its active site

Since PPIA stimulation of IL-8 appears to be indepen-dent of cellular BSG in at least one monocytic cell line (Figure 5C), we sought a direct confirmation of extracellular PPIA binding to heparin, which is the other proposed cellular target of extracellular PPIA [1]. In fact, other cyclophilins such as cyclophilin-B (CypB) also engage heparin [43], yet this has not directly been shown for PPIA at atomic resolution and a confirmation would potentially set the stage for the search for other glycoprotein receptors beyond BSG. Since we have previously shown that PPIA both binds and catalyzes a single site within BSG that is adjacent to the outer cellular membrane by NMR [17], here we also employed NMR to probe the potential PPIA/heparin interaction. NMR is an ideal technique for probing low affinity interactions such as those between PPIA and its targets. For example, the PPIA/BSG interaction is an extremely low affinity interaction that we have characterized by NMR [17] and others have characterized low affinity intracellular PPIA interactions using NMR as well [16].

**Figure 6:**
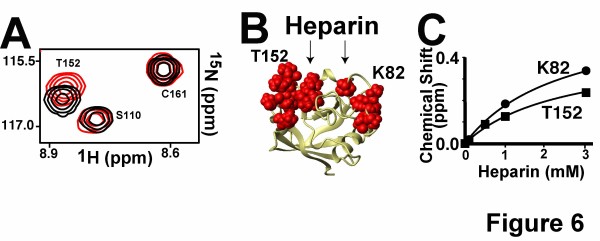
**Quantifying the PPIA interaction with heparin at atomic resolution. A**) ^15^N-HSQC spectra of 0.5 mM ^15^N-PPIA alone (black) and in complex with 3 mM heparin (red). **B**) Residues that exhibit chemical shifts greater than 0.1 ppm upon engaging heparin in either ^15^N or ^1^H dimensions are highlighted (red) and are all located within the PPIA active site. **C**) Based on the measured ^15^N-PPIA chemical shift changes upon titration with heparin, the binding isotherms were used to estimate a K_D_ of 2.5 ± 0.3 mM. Shown are the binding isotherms for the ^15^N chemical shift changes of K82 and T152. All experiments were conducted at 600 MHz at 25°C.

The titration of heparin into a solution of recombinant ^15^N-labeled PPIA induced specific chemical shift changes as monitored through ^15^N-HSQC spectra (Figure 6A). Only active site residues exhibited chemical shift changes (Figure 6B), indicating that heparin interactions are specific to this region. Amides that exhibit chemical shift changes above the digital resolution of the indirect dimension (~0.1 ppm) include H54, R55, I56, G59, S77, G80, K82, N102, N108, S147, N149, K151, T152, S153, and K155. An estimate for the dissociation constant (K_D_) of PPIA/heparin was calculated as 2.5 ± 0.3 mM utilizing these chemical shift changes with a simultaneous fit to a simple binding isotherm (Figure 6C). Such weak affinity is on the order of that also determined in vitro for the PPIA/BSG interaction [17]. However, it should be noted that the commercially available heparin is a mixture of different lengths and thus, the specific affinity to one particular heparin may be much higher. Regardless, our findings indicate that PPIA does indeed target heparin specifically through its active site as previously postulated [44].

## Discussion

Using both epithelial cells and monocytic cells, we have identified both similar and markedly different responses to exogenous PPIA. Several of the key new findings include the following: i) The biological activity of PPIA was partially abrogated by a PPIA active site mutant (Figure 2), thereby both confirming specificity and suggesting that the PPIA active site is important for some of its many biological activities. However, the important and potentially novel finding here is that the PPIA active site mutant still retains a significant residual biological activity, despite the fact that this mutant is reported to be only 1 % as enzymatically active as the wild-type [32]. This suggests that the PPIase activity of PPIA may not be important for all of its extracellular functions, which is analogous to recent findings that have suggested the PPIase activity is not required for all of its intracellular functions [45,46]. ii) Similarities were identified in response to PPIA, which included the production of IL-8 and p21 (Figure 1) as well as activation of NFκB (Figure 3). In fact, a novel finding here is that the PPIA-mediated response for several cytokines is completely reliant on the downstream activity of NFκB and consistent with this, all cell lines also showed translocation of NFκB from the cytoplasm to the nucleus upon PPIA stimulation (Figure 3). These similarities may suggest that extracellular PPIA contributes to the relatively high concentrations of key factors such as IL-8 that drive cancer progression [27] and p21 levels that have also been found to correlate to the progression of pancreatic cancer [24]. iii) Several differential responses were identified in response to PPIA. These cell-dependent responses were observed in regard to PPIA stimulation of ERK1/2 phosphorylation, indicating that the signaling pathways that lead to NFκB translation are cell-dependent. Another novel finding here is that there is a differential response observed for cell lines even derived from the same cancer. Specifically, the two pancreatic cell lines, PANC-1 and L3.6pL, exhibit a markedly different response to extracellular PPIA with regard to IL-5 and IL-6 (Figure 1C,D) as well as ERK1/2 phosphorylation (Figure 4). Differential responses to exogenous PPIA also hold true for the monocytic cell lines used here as well, MOLM13 and U937 cells. Specifically, despite the common myeloid lineage of both cell lines, extracellular PPIA only induced cell surface expression of BSG within MOLM13 cells, but not in U937 cells (Figure 5B,C). Nonetheless, our identification of PPIA-induced cellular BSG expression has important implications for cancer progression in regard to the relationships between PPIA, BSG, and MMPs. Namely, our data show that in some cell types, PPIA stimulates cellular BSG expression, which is in turn a known regulator of many MMPs that drive tumorigenesis [47]. iv) Extracellular PPIA was not necessarily reliant on cellular BSG for all of its activities (Figure 5). For example, knockdown of the cellular BSG in monocytic cells resulted in abrogation of PPIA-mediated ERK1/2 phosphorylation. However, knockdown of cellular BSG did not result in a detectable change in PPIA-mediated stimulation of IL-8, suggesting that BSG is not the sole cellular target of extracellular PPIA and, therefore, opening avenues for the further identification of PPIA cellular receptors.

The identification of cellular BSG as a target for extracellular PPIA has greatly enhanced the field of PPIA-mediated signaling [6], yet studies such as those conducted here reveal that PPIA signaling is far more complex than previously thought. Specifically, extracellular PPIA likely interacts with multiple cellular receptors. For example, our previous atomic resolution investigations with PPIA and BSG revealed that these two proteins bind with millimolar affinity and therefore may require other mediators to enhance their biological interactions [17]. This is supported by our studies here that have shown recombinant PPIA is biologically active at much lower concentrations that are within the micromolar range, which is more reflective of those concentrations that have been detected in vivo [10]. More importantly, the very fact that BSG is necessary for PPIA stimulation of ERK1/2 phosphorylation (in MOLM13 cells in Figure 5A), but dispensable for IL-8 stimulation (in U937 cells in Figure 5C) could imply that there is at least one more cellular receptor beyond BSG. This is further supported by the differential cellular response imparted by extracellular PPIA (Figure 1 and Figure 4), which may not be surprising in retrospect. For example, intracellular PPIA interacts with numerous targets that include intracellular regions of receptors such as prolactin [48] along with multiple kinases [16]. Thus, extracellular PPIA has likely also evolved to interact with multiple extracellular targets thereby regulating many cellular responses that are simply dependent on expressed receptors. In fact, extracellular PPIA has recently been shown to mediate polymerization of the extracellular protein hensin [49], which in turn mediates assembly of the extracellular matrix. Previous studies using PANC-1 cells have shown that PPIA-induction of IL-17 is only partially reduced upon blocking cellular BSG, also suggesting that other cellular receptors are involved in IL-17 stimulation [19]. All of these findings have critical implications to a field where BSG has been thought to be the only cellular receptor. Unfortunately the identification of PPIA targets has been met with limited success (both extracellular and intracellular), which is likely due to the transient nature of PPIA interactions that makes simple pull-down experiments difficult. Future studies aimed at identifying the other cellular receptors of PPIA will likely require recently developed approaches that are more directed at low affinity interactions such as crosslinking methods [50,51]. Thus, hensin and BSG already provide an initial short list of the multiple proteins that extracellular PPIA engages, yet several studies including ours here also suggest that glycosaminoglycans or other potential cellular protein receptors serve as other cellular targets. Our confirmation that PPIA engages one such glycosaminoglycan directly through its active site, i.e., heparin (Figure 6), supports several previous studies that have indicated that these glycans serve as the initial interactions of extracellular cyclophilins with the cell surface [1,43,44,52].

## Conclusions

The major outcome from the studies conducted here is that extracellular PPIA induces cell-specific responses that are not all reliant on cellular BSG. These cell-specific responses have important implications for the rationale design of therapeutic strategies aimed at blocking extracellular PPIA functions during disease progression. Namely, our studies suggest that the initial cellular targets and associated signaling events differ significantly between cells and reveal the importance of characterizing extracellular PPIA functions within multiple cell lines. In general, the notion of one ligand targeting one receptor is a rare phenomenon (i.e., PPIA solely targeting the cellular BSG receptor). The very fact that extracellular PPIA has been compared to cytokines that each have multiple receptors also suggests that PPIA targets multiple cellular receptors that may in turn be expressed in a cell type-dependent manner.

## Methods

### Protein expression and purification

All proteins encoded in pET vectors were transformed into BL21/DE3 cells for subsequent growth and purification. Wild type PPIA and a PPIA R55A mutant were purified through a three-column purification as previously described [17]. The DNA encoding IL-6 and IL-8 were purchased from Open Biosystems (Huntsville, AL), cloned into a pET15b vector and the mature forms were expressed, refolded, and purified as we have previously described for several proteins [17,53]. Endotoxin levels were monitored by ToxinSensor Chromogenic LAL Endotoxin Assay (GenScript). The final purified proteins were concentrated to 1–2 mM using Millipore concentrator and stored at −80°C until use. PPIA and its mutant were diluted in 50 mM phosphate buffer, pH 6.5 while both interleukins were diluted to the same buffer but with an additional 150 mM NaCl. These buffers were selected since repeated cycles of freeze/thawing produced no visible precipitate. We note that any additional salt added to recombinant PPIA did result in precipitation upon freeze/thawing. All proteins were sterilized prior to use in cell-based assays.

### Cell culture

HEK293T, MOLM13, and U937 cells were a kind give from Dr. Christopher C. Porter (Department of Pediatrics, University of Colorado Denver). L3.6pL cells were a kind gift from Dr. Isaiah J. Fidler, Department of Cancer Biology, The University of Texas MD Anderson Cancer Center). PANC-1 cells were purchased from ATCC (ATCC Number CRL-1469). HEK293T, PANC-1, and L3.6pL and cells were cultured in Dulbecco’s modified Eagle medium with 10 % fetal bovine serum (FBS) at 37°C with 5 % CO_2_. MOLM13 and U937 cells were cultured in RPMI 1640 medium with 10 % fetal bovine serum at 37°C with 5 % CO_2_. Fresh media was replaced every 2 to 3 days. Under the concentrations of PPIA utilized in this study, no cell death was observed.

### Luciferase reporter assays

Luciferase reporter plasmids are described in Additional file 5. In addition to the previously constructed mutations to the NFκB binding element in the pGL3-IL-6 reporter [38], similar mutations were made here within the pGL3-IL-8 reporter. Site directed mutagenesis was used to construct each mutation, which were then confirmed by sequencing. Specifically, the wild type NFκB binding site within the IL-8 promoter comprises the following sequence that was used as a template for site-directed mutagenesis: 5’-TCAGTTGCAAATCGTGGAATTTCCTCTGACATAATG-3’. The following primers and their reverse complements were used to create pGL3-IL-8 Mut1 and Mut2 respectively: 5’-TCAGTTGCAAATCGTTAACTTTCCTCTGACATAATG-3’ (Mut1) and 5’-TCAGTTGCAAATCGTGCAATGTCGTCTGACATAATG-3’ (Mut2).

For transfections of luciferase reporters, cells (1 × 10^5^) were plated into 6-well plates and cultured for 1 day in complete medium, then transfected with 6 μg per well of reporter plasmids and 25 ng per well of Renilla luciferase plasmid, using Turbofact (Fermentas). 24 h post-transfection, cells were serum starved for 4 h and then stimulated with the indicated concentrations of recombinant proteins or buffer (as control) in media without FBS for another 24 h. Firefly and Renilla luciferase activity were measured with the dual-luciferase reporter assay system (Promega) and the former was normalized to the latter to account for transfection efficiencies. Finally, results are presented as the fold-increase of cells (Luc Fold-Increase), which is the ratio of cells treated with recombinant protein over cells treated with the same amount of buffer control.

### NFkB translocation

HEK293T, PANC-1, and L3.6pL cells were cultured on glass coverslips in 6 well plates. To detect NFκB translocation from the cytoplasm to the nucleus, these cells were transfected with 6 μg of a plasmid encoding for p65 and GFP for 24 h as described previously [54], which was a kind gift from Dr. M. Lienhard Schmitz, University of Bern, Department for Chemistry and Biochemistry, Bern, Switzerland. Cells were then stimulated with 25 μM PPIA for 24 h and buffer was used as a control. Hoechst 33342 dye (Sigma, St. Louis, MO) was used for nuclear staining (blue). Cells were washed with PBS, fixed with cold methanol for 10 min at 4°C, and analyzed using a fluorescence microscope (Zeiss).

### Western blotting, immunocytofluorescence and flow cytometric analysis

For protein detection by western blot analysis, whole-cell lysates were harvested and lysed for 30 min in ice using 100 μL of RIPA buffer. The lysates were cleared via centrifugation at 13,000 rpm for 10 min in a microcentrifuge. Supernatants were collected and then subjected to western blotting with antibodies to unphosphorylated total ERK1/2 (ERK1/2) and phosphorylated ERK1/2 (pERK1/2) both purchased from the same source (Cell Signaling) or antibodies to BSG (R&D Systems). Ten μL of total lysate was subjected to electrophoresis on SDS-PAGE gels and transferred to a PVDF membrane. The membranes were blocked and incubated with primary and secondary antibodies according to the manufacturer’s protocols. Signals were developed using western LightningR Plus-ECL Enhanced Chemiluminescent Substrate (PerkinElmer, Waltham, MA) and exposed to X-ray film.

To assess ERK1/2 phosphorylation using flow cytometry, cells were cultured in 6-well plates at approximately 1x10^5^ per well and treated with either PPIA at 25 μM or buffer for the indicated times. The ERK1/2 phosphorylation assay was quantified by flow cytometry using anti-pERK1/2 (BD Biosciences) performed as we have previously described [55].

For IkBα degradation, PANC-1, L3.6pL and HEK293T were treated with 25 μM recombinant PPIA for 2 h. Cells were fixed with methanol for 10 min at 4°C and blocked with 3 % normal donkey serum (Jackson ImmunoResearch; West Grove, PA) in PBS for 1 h. The cells were incubated with rabbit IkBα antibody (Santa Cruz Biotechnology Inc., Santa Cruz, CA) for 1 h at room temperature. The secondary antibody, Alexa Fluor 594 anti-rabbit IgG (Invitrogen Corp., Carlsbad, CA), was applied for 1 h. Cells were mounted with Vectashield medium containing DAPI (Vector Laboratories, Burlingame, CA).

### BSG knockdown in monocytes

Commercially available BSG shRNA lentiviral vectors (pLKO.1 plasmids purchased from OpenBiosystems, Huntsville, AL) were transfected into HEK293FT cells at 70 %-80 % confluency with 500 ng of the necessary viral packaging vectors using TurboFect (Fermentas). Viral packaging vectors included REV, GAG/Pol, and VSVG. One mL of the supernatants comprising the viruses produced from these HEK293FT cells were then transduced using 5x10^4^ MOLM13 or U937 cells in the presence of polybrene (Sigma) and puromycin resistance encoded within the lentiviral shRNA was used to select for successful transductions. A substantial BSG knockdown for both MOLM13 and U937 cell lines was found for an BSG shRNA that targets the 3’-UTR of the non coding region of BSG, herein called shBSG, and was utilized for all knockdowns described. A non-encoding shRNA purchased from Addgene (Cambridge, MA), herein called sh0, was used as a control.

### Cell cycle analysis

Cells were plated in 6 well plates for 24 h, followed by starvation for 4 h and then stimulated with either 25 μM PPIA or buffer for 24 h. After stimulation, cells were stained overnight at 4°C with a solution containing 25 μg/mL propidium iodide, 0.3 % saponin, 0.1 mM EDTA, and 2 Kunitz U/mL RNAse. The cells were analyzed for a sub-G1 peak using a Coulter Epics XL flow cytometer (Beckman-Coulter, Hialeah, Florida). Peak vs. integral gating was used to exclude doublet events from the analysis. Data was collected for 10,000 cells. Modfit LT (Verity Software House, Topsham, Maine) was used for cell cycle and apoptotic peak modeling.

### NMR sample preparation and experiments

ZZ-exchange spectroscopy was used for assessing the catalytic activity of both wild type PPIA and the PPIA R55A mutant. ZZ-exchange spectroscopy was performed on a Varian 720 at 10°C using 20 μM enzyme and 1 mM of a recombinantly produced ^15^ N-labeled peptide substrate with the following resultant sequence: GSFGPDLRAGD. This peptide was produced and its resonances assigned as previously described by Schlegel et al. [17]. A ^15^ N-edited heteronuclear single quantum coherence (^15^ N-HSQC) was also collected on the free peptide substrate alone. NMR buffer conditions were 50 mM NaPO_4_, pH 6.5, 1 mM dithiothreitol with 5 % D_2_O. The resonance shifts of the model peptide substrate were assigned via standard triple resonance experiments as we have previously described for proteins [17,53,56].

For assessing PPIA binding to heparin, PPIA was ^15^ N-labeled and ^15^ N-HSQC spectra were collected on a Varian 600 with increasing amounts of heparin derived from porcine intestinal mucosa (Sigma, catalogue number 9041-08-1). The concentration of ^15^ N-labeled PPIA was 0.5 mM and final concentrations of heparin added were 0, 100, 500, 1000, 3000 μM. NMR buffer conditions were 50 mM HEPES, pH 7.5, 1 mM dithiothritol with 5 % D_2_O.

### Statistical analysis

One-way ANOVA by GraphPad Prism 5 was used to evaluate statistical differences among experimental groups. A Dunnett's test was applied and a value of *p* < 0.05 was considered significant (*). Data are shown here as the mean ± SEM from three independent experiments.

## Abbreviations

PPIA: Cyclophilin-A; BSG: Extracellular Matrix MetalloPRoteinase Inducer; GFP: Green Fluorescent Protein; IL: Interleukin; MMP: Matrix Metalloproteinase; NFκB: Nuclear Factor Kappa B.

## Competing interests

There are no competing interests.

## Authors’ contributions

KB designed and acquired all of the cell-based assays, MH and FZ produced and collected the nuclear magnetic resonance experiments. CH, JD, and RAS helped design and acquire the ERK1/2 phosphorylation experiments and provided expertise in culturing monocytic cell lines. JD and JR helped produce stable knockdown cell lines. MC and CW provided expertise in culturing pancreatic cancer cell lines. EZE helped design all of the experiments. All authors read and approved the final manuscript.

## Supplementary Material

Additional file 1 **Figure S1.** Assessing the purity of recombinant PPIA. A) UV spectrum of recombinantly purified PPIA. The atypical UV spectrum of PPIA has been shown to be an important confirmation of its purity [15]. B) ^15^N-HSQC spectrum of purified PPIA collected at 900 MHz at 25°C along with the three-dimensional structure (inset). Click here for file

Additional file 2 **Figure S2.** The effect of extracellular PPIA on cellular proliferation. Cell cycle was monitored 24 h post incubation using FACS analysis with either buffer alone or recombinant PPIA in (A) HEK293T cells, (B) MOLM13 cells, (C) PANC-1 cells, and (D) L3.6pL cells. No apparent effect was observed. Click here for file

Additional file 3 **Figure S3.** Probing the cell-specific responses to in-house purified recombinant proteins. A) MOLM13 cells were used for a comparative analysis of luciferase reporter assays stimulated with various recombinantly purified proteins, which include recombinant PPIA and recombinant IL-6. B) Luciferase reporter activity of PPIA was monitored for both IL-6 (left) and IL-8 (right). All luciferase reporter activities were conducted as in Figure 1. Click here for file

Additional file 4 **Figure S4.** Further characterization of PPIA-mediated activation of NFκB. A) An NFκB translocation assay was conducted as in Figure 3A, but using the PPIA active site point mutation, PPIA R55A. B) IκBα degradation is shown after treatment with recombinant PPIA. Click here for file

Additional file 5 Luciferase Reporter Plasmid Sources. Click here for file
