# Supplementary figures and images for: Extracellular cyclophilin-A stimulates ERK1/2 phosphorylation in a cell-dependent manner but broadly stimulates nuclear factor kappa B
Source: Cancer Cell Int. 2012 Jul 4;12:19. doi: 10.1186/1475-2867-12-19 (PMC3390265; doi:10.1186/1475-2867-12-19)

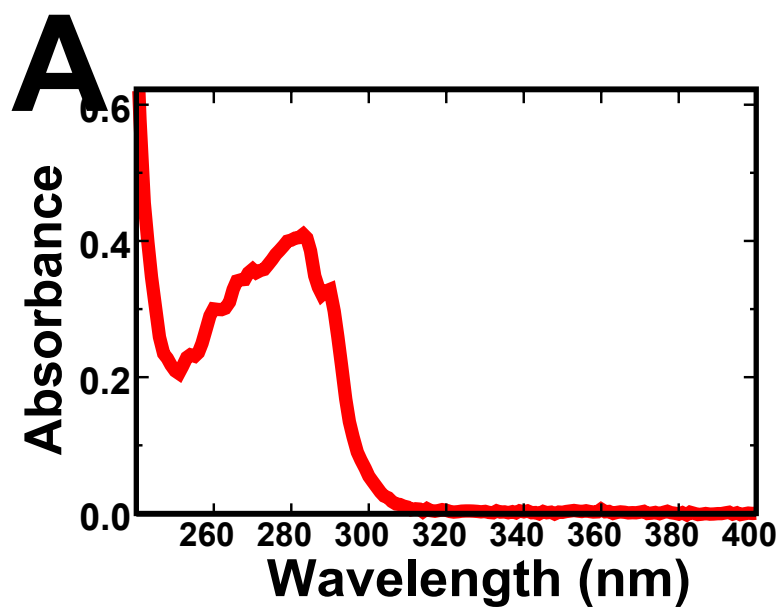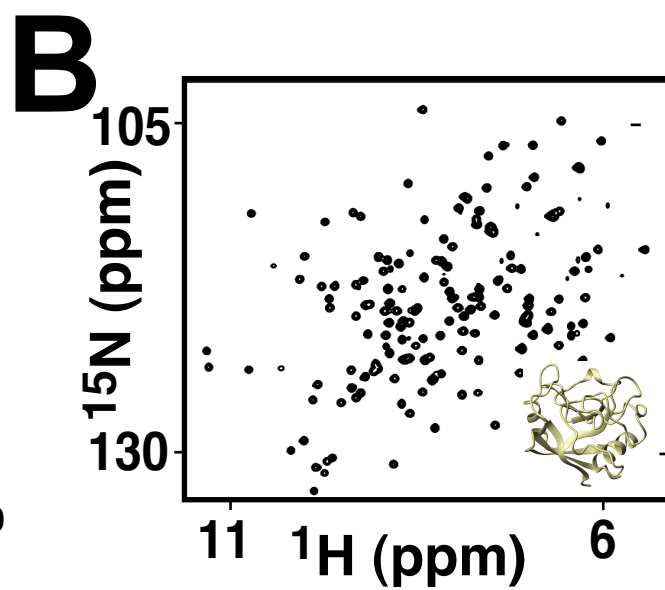

**Figure S1**

Supplement: Additional file 1 — Figure S1. Assessing the purity of recombinant PPIA. A) UV spectrum of recombinantly purified PPIA. The atypical UV spectrum of PPIA has been shown to be an important confirmation of its purity [15]. B) 15N-HSQC spectrum of purified PPIA collected at 900 MHz at 25°C along with the three-dimensional structure (inset). [file 1475-2867-12-19-S1.pdf]

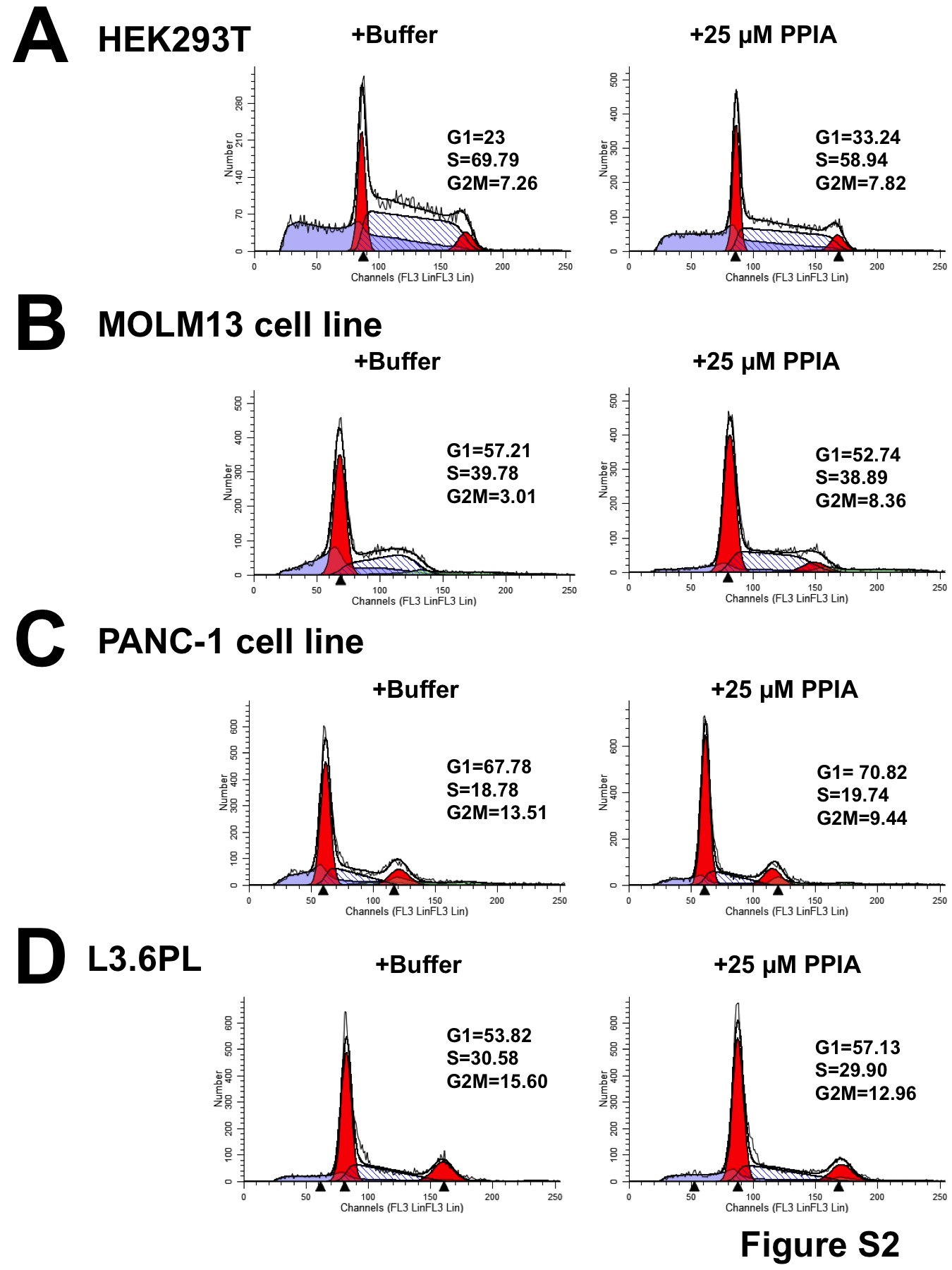

Supplement: Additional file 2 — Figure S2. The effect of extracellular PPIA on cellular proliferation. Cell cycle was monitored 24 h post incubation using FACS analysis with either buffer alone or recombinant PPIA in (A) HEK293T cells, (B) MOLM13 cells, (C) PANC-1 cells, and (D) L3.6pL cells. No apparent effect was observed. [file 1475-2867-12-19-S2.jpeg]

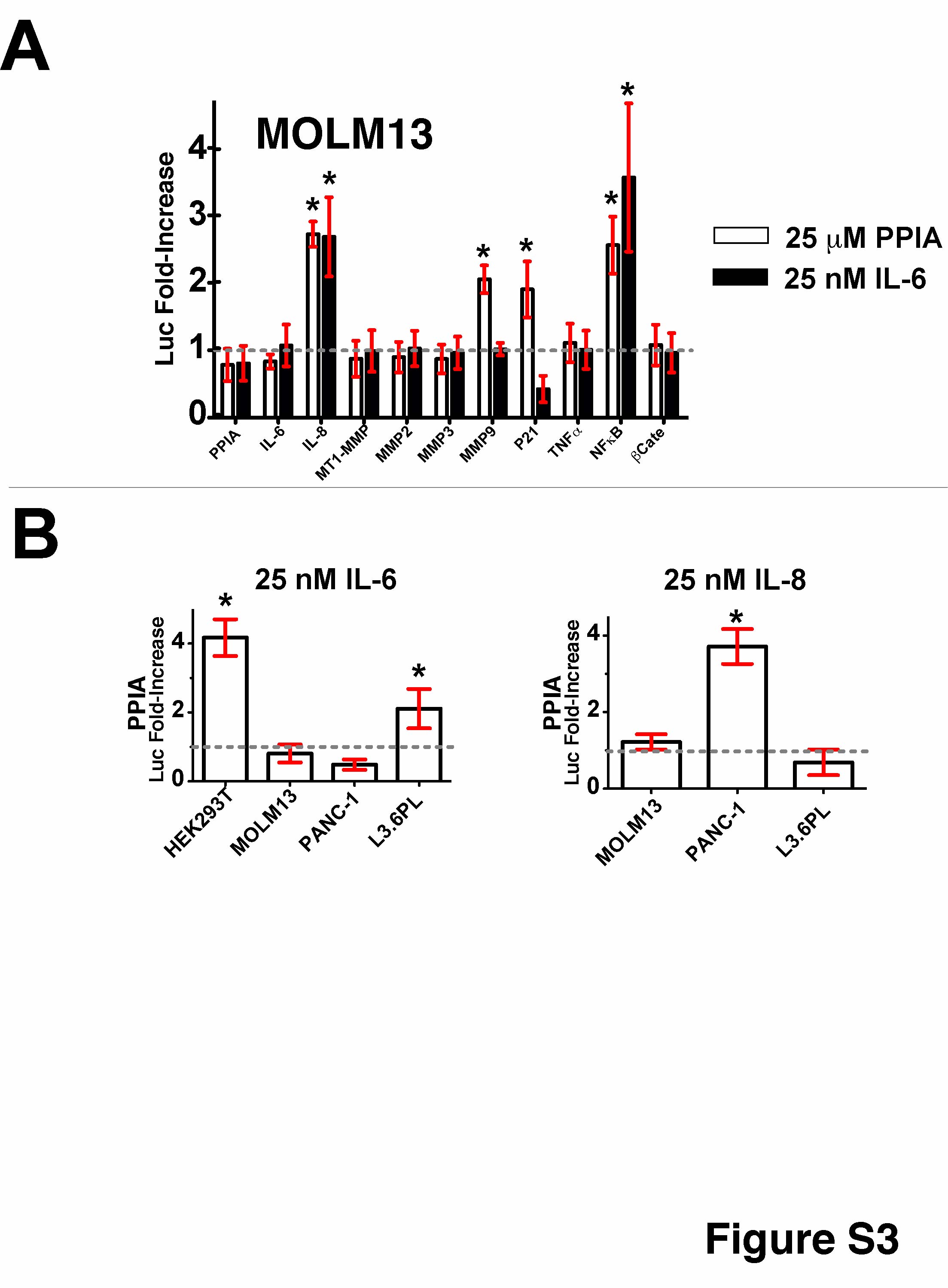

Supplement: Additional file 3 — Figure S3. Probing the cell-specific responses to in-house purified recombinant proteins. A) MOLM13 cells were used for a comparative analysis of luciferase reporter assays stimulated with various recombinantly purified proteins, which include recombinant PPIA and recombinant IL-6. B) Luciferase reporter activity of PPIA was monitored for both IL-6 (left) and IL-8 (right). All luciferase reporter activities were conducted as in Figure 1. [file 1475-2867-12-19-S3.jpeg]

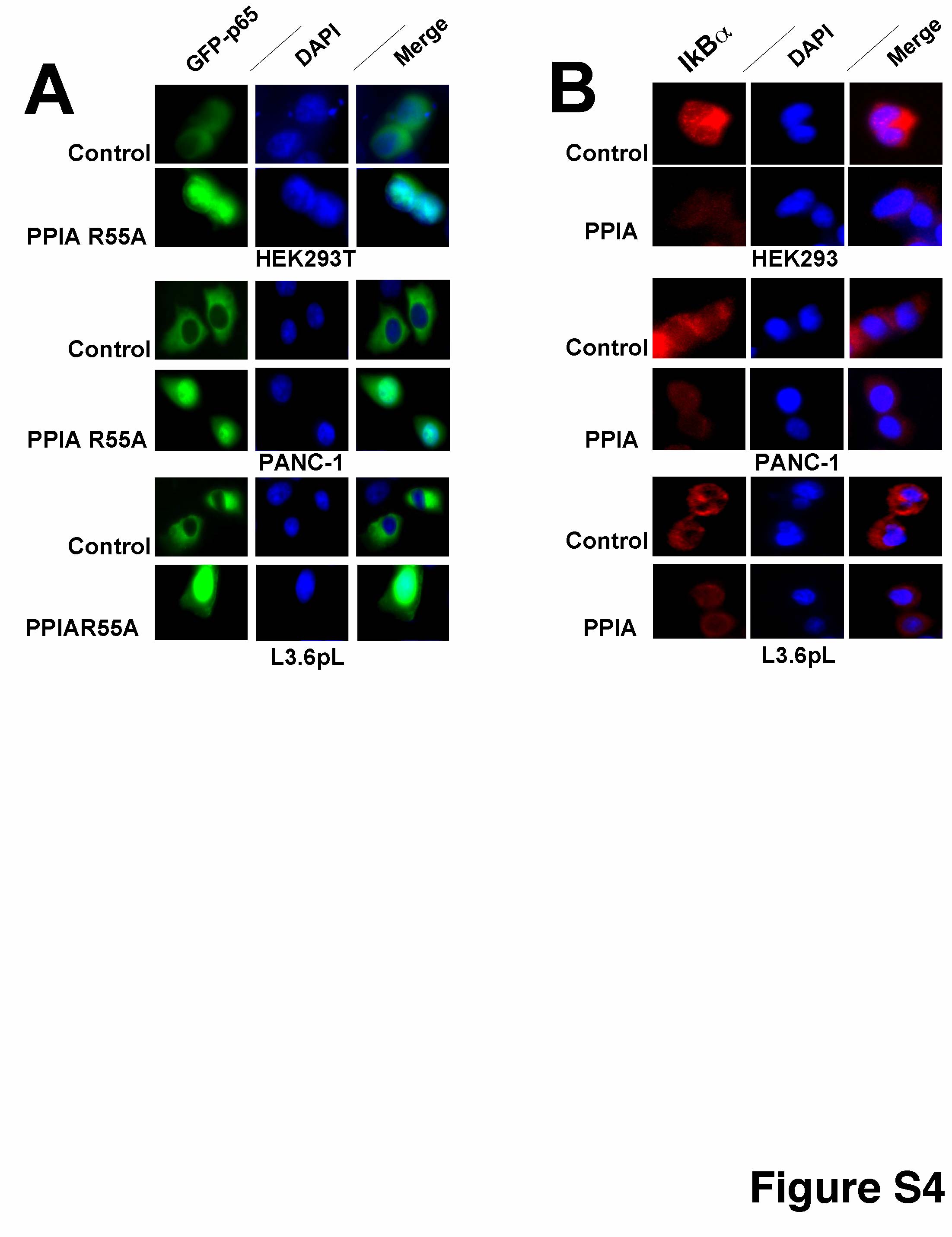

Supplement: Additional file 4 — Figure S4. Further characterization of PPIA-mediated activation of NFκB. A) An NFκB translocation assay was conducted as in Figure 3A, but using the PPIA active site point mutation, PPIA R55A. B) IκBα degradation is shown after treatment with recombinant PPIA. [file 1475-2867-12-19-S4.jpeg]
