# Supplementary material for: Extracellular cyclophilin-A stimulates ERK1/2 phosphorylation in a cell-dependent manner but broadly stimulates nuclear factor kappa B
Source: Cancer Cell Int. 2012 Jul 4;12:19. doi: 10.1186/1475-2867-12-19 (PMC3390265; doi:10.1186/1475-2867-12-19)
Supplement: Additional file 5 — Luciferase Reporter Plasmid Sources. [file 1475-2867-12-19-S5.doc]

Luciferase Reporter Plasmid Sources.

Luciferase reporter plasmids were kind gifts from the following sources: pGL3-IL-8 and pGL3-IL-2 were obtained from Dr. James Lambert, Department of Pathology, University of Colorado Denver. pGL3-VEGFC, pGL3- NFκB and the TOPFlash vector comprising multiple β-catenin transcription sites were obtained from Dr. Heide Ford,  Department, Obstetrics and Gynecology, University of Colorado Denver. pGL3-p21^CIP1^, referred to here as p21, was obtained from Dr. Ken-ichi Isobe, Department of Basic Gerontology, National Institute for Longevity Sciences, Aichi, Japan {Xiao, 1999 #1353}. pGL3–HRE was obtained  from Dr. Sean  P. Colgan,  Department of Medicine, University of Colorado Denver. PGL3-MMP2 and pGL3-MMP3 were obtained from Dr. Weston Porter, Department of Veterinary Integrative Biosciences, Texas A&M University. PGL3-PPIA was obtained from Dr. Sung Soo Kim, Department of Biochemistry and Molecular Biology, Kyung Hee University, Korea. PGL3-MT1-MMP1 was obtained from Dr. G. Timothy Bowden, Department of Molecular and Cellular Biology, University of Arizona. PGL3-TNFα was obtained from Dr. Laura Nagy, Department of Pathobiology, Lerner Research Institute – The Cleveland Clinic Foundation. PGL2-IL-5 was obtained from Dr. Sandra Stevenson, School of Biomedical Sciences Molecular Immunology Group, Health Sciences Curtin University, Australia. pGL3-MMP9 was obtained from Dr. Douglas D. Boyd, Department of Cancer Biology, M.D. Anderson Cancer Center. pGL3-BSG was obtained from Dr. Luigi Puglielli, Department of Medicine, University of Wisconsin. All pGL3-IL-6 reporters that included the wild type promoter and two previously described mutations to the NFκB binding element, Mut1 and Mut2 {Schwaninger, 1999 #1187}, were obtained from Dr. Markus Schwaninger, Institute of Experimental and Clinical Pharmacology and Toxicology, University of Lübeck, Germany.
